# Mitochondrial chaperon TNF-receptor- associated protein 1 as a novel apoptotic regulator conferring susceptibility to *Pneumocystis jirovecii pneumonia*

**DOI:** 10.3389/fimmu.2024.1423086

**Published:** 2024-08-19

**Authors:** Aseervatham Anusha Amali, Kathirvel Paramasivam, Chiung Hui Huang, Abhinav Joshi, Jayshree L. Hirpara, Sharada Ravikumar, Qi Hui Sam, Rachel Ying Min Tan, Zhaohong Tan, Dilip Kumar, Leonard M. Neckers, Shazib Pervaiz, Roger Foo, Candice Y Y Chan, Jin Zhu, Cheryl Lee, Louis Yi Ann Chai

**Affiliations:** ^1^ Division of Infectious Diseases, Department of Medicine, National University Health System, Singapore, Singapore; ^2^ Mechanobiology Institute, National University of Singapore, Singapore, Singapore; ^3^ Department of Paediatrics, Khoo Teck Puat – National University Children’s Medical Institute, Singapore, Singapore; ^4^ Urologic Oncology Branch, Center for Cancer Research, National Cancer Institute (NCI), Bethesda, MD, United States; ^5^ Cancer Science Institute, National University of Singapore, Singapore, Singapore; ^6^ Synthetic Biology for Clinical and Technological Innovation (SynCTI), National University of Singapore, Singapore, Singapore; ^7^ Singapore Immunology Network (SIgN), ASTAR (Agency for Science, Technology and Research), Singapore, Singapore; ^8^ Department of Physiology, Yong Loo Lin School of Medicine, National University of Singapore, Singapore, Singapore; ^9^ Cardiovascular Research Institute, National University Health System, Singapore, Singapore; ^10^ Department of Medicine, Yong Loo Lin School of Medicine, National University of Singapore, Singapore, Singapore; ^11^ Department of Infectious Diseases, Singapore General Hospital, Singapore, Singapore; ^12^ Cardiovascular and Metabolic Disorders, Duke NUS Medical School, Singapore, Singapore

**Keywords:** opportunistic infection, mitochondria, oxidative phosphorylation, glycolysis, caspase, apoptosis

## Abstract

Molecular chaperons stabilize protein folding and play a vital role in maintaining tissue homeostasis. To this intent, mitochondrial molecular chaperons may be involved in the regulation of oxidative phosphorylation and apoptosis during stress events such as infections. However, specific human infectious diseases relatable to defects in molecular chaperons have yet to be identified. To this end, we performed whole exome sequencing and functional immune assessment in a previously healthy Asian female, who experienced severe respiratory failure due to *Pneumocystis jiroveci pneumonia* and non-HIV-related CD4 lymphocytopenia. This revealed that a chaperon, the mitochondrial paralog of HSP90, TRAP1, may have been involved in the patient’s susceptibility to an opportunistic infection. Two rare heterozygous variants in TRAP1, E93Q, and A64T were detected. The patient’s peripheral blood mononuclear cells displayed diminished TRAP1 expression, but had increased active, cleaved caspase-3, caspase-7, and elevated IL-1β production. Transfection of A64T and E93Q variants in cell lines yielded decreased TRAP1 compared to transfected wildtype *TRAP1* and re-capitulated the immunotypic phenotype of enhanced caspase-3 and caspase-7 activity. When infected with live *P. jiroveci*, the E93Q or A64T TRAP1 mutant expressing cells also exhibited reduced viability. Patient cells and cell lines transfected with the TRAP1 E93Q/A64T mutants had impaired respiration, glycolysis, and increased ROS production. Of note, co-expression of E93Q/A64T double mutants caused more functional aberration than either mutant singly. Taken together, our study uncovered a previously unrecognized role of TRAP1 in CD4^+^ lymphocytopenia, conferring susceptibility to opportunistic infections.

## Introduction

*Pneumocystis jirovecii pneumonia* (PjP) is a well-recognized manifestation of advanced HIV disease and as an opportunistic pathogen is seen in immunocompromised patients in haemato-oncology and transplantation. Beyond this, less is understood about other intrinsic host immunity factors dictating susceptibility to PjP infection (1).

Mitochondrial chaperons, through their ability to fold or disaggregate cellular protein assembly, are important in maintaining healthy mitochondrial functions such as oxidative phosphorylation and apoptosis (2). Reactive oxygen species (ROS), as products of oxidative phosphorylation and apoptosis, are critical in the suppression of infections through the elimination of pathogens and infected cells (3). Conversely, dysregulated mitochondrial chaperoning can result in excessive ROS and cell death that exacerbate tissue damage and impaired leukocyte function. Through a case of idiopathic CD4^+^ lymphopenia, we uncovered a previously unrecognized role of the mitochondrial chaperon protein Tumour Necrosis Factor-Receptor Associated Protein 1 (TRAP1) in CD4^+^ lymphocytopenia and in the mechanism of PjP immunopathogenesis. This investigation sheds light on the involvement of mitochondrial chaperons in immunity and susceptibility to infection.

## Results

### Clinical setting

The investigations arose from the proband, a 48-year-old female of Asian origin who was previously well other than a history of schizophrenia, presenting to the hospital with 6 months of persistent cough and worsening breathlessness. Chest imaging revealed diffuse ground glass opacities. The patient rapidly deteriorated and required intubation for severe type I respiratory failure requiring veno-venous extra-corporeal membrane oxygenation assistance. The clinical course of the patient is as described in **Supplementary Figure S1A**. Bronchoalveolar lavage examination revealed *Pneumocystis jirovecii* on microscopy. Treatment with trimethoprim-cotrimoxazole (TMP-SMX) and adjunctive corticosteroids was promptly initiated. Despite repeat negative HIV tests, the patient had an absolute CD4^+^ count of 25 cells/µl. Over 6 weeks, she progressively improved before discharge 3 months later. As her CD4^+^ counts had remained consistently low, the patient was prescribed long-term TMP-SMX as a secondary prophylaxis and remained stable.

### Whole exome sequencing revealed two SNPs in the functional domain of TRAP1

**Figure 1:**
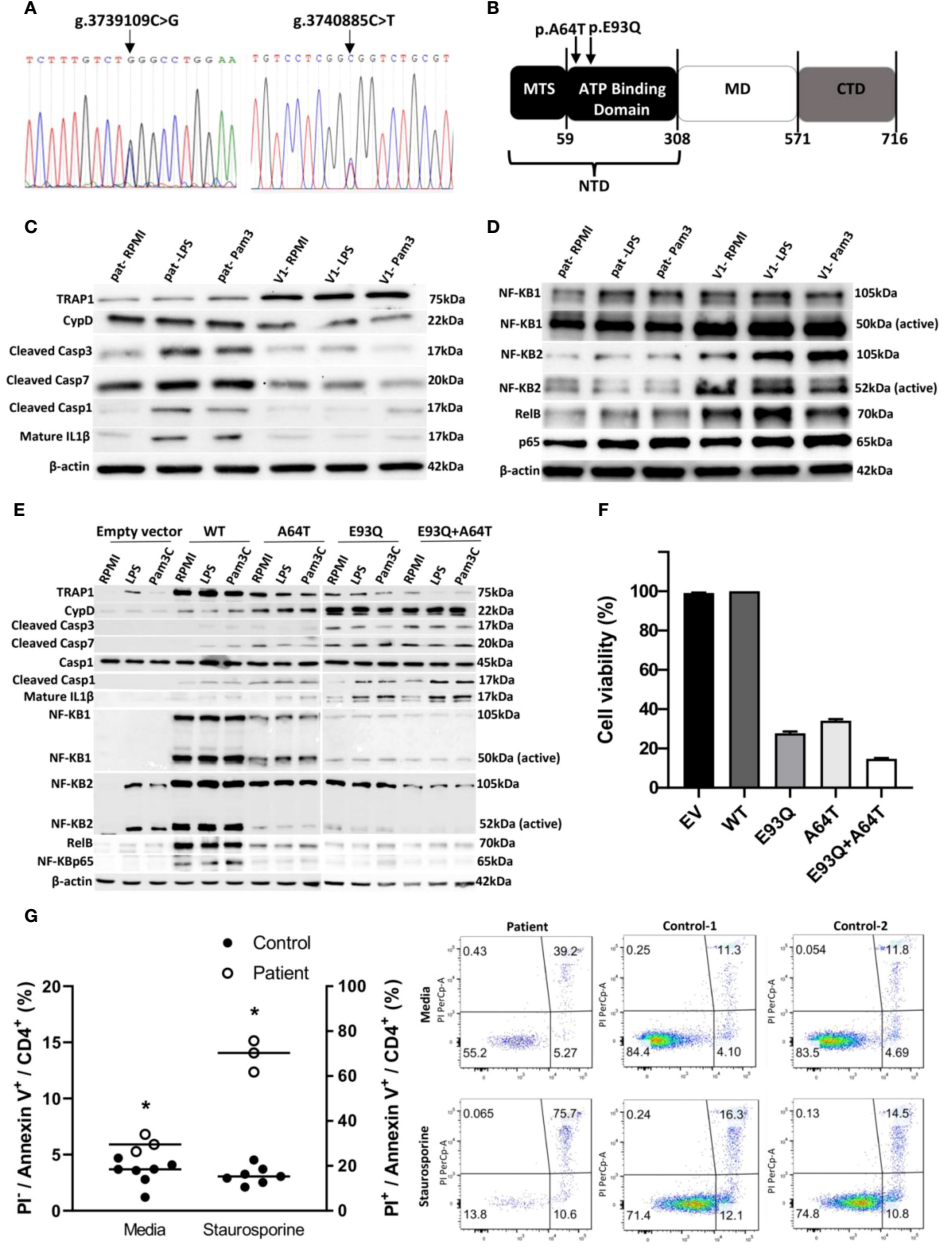
TRAP1 mutation increased caspase activity *in vitro* and *in vivo* and inflammasome activation, resulting in enhanced apoptosis. **(A)** Sanger sequencing electropherograms of patient genetic variants. **(B)** Schematic representation of the domain structure of Trap1. Trap1 has three structural domains, an N-terminal domain (NTD) containing mitochondria targeting sequence (MTS) and an ATPase domain, a middle domain (MD) responsible for client binding, and a C-terminal domain (CTD) for dimerization. The arrows indicate the location of the amino acid switch from Glutamate to Glutamine (p.E93Q) and alanine to threonine switch at the position of 64 (p.A64T) in Trap1. **(C, D)** Western blot of peripheral blood mononuclear cells (PBMCs) from the patient and control was stimulated with LPS and Pam3C or unstimulated (RPMI) for 30 minutes. Cell lysates were run on a 10% SDS gel, transferred to PVDF membrane, and probed for different antibodies. β-actin was used as a loading control. **(E)** A549 cells were transfected with empty vector, Trap1 wild-type (WT), Trap1 p.A64T (A64T), Trap1 p.E93Q (E93Q), and Trap1 p.A64T+Trap1 p.E93Q (A64T+E93Q) and were stimulated with LPS and Pam3C and the cell lysates were analyzed by Western blot. All Western blots were repeated three times. **(F)** PCP infection inhibits cell viability in A549 Trap1 transfected cells. The cell viability was measured using the XTT Assay. Values are the mean 3 ± SEM from nine replicates of three independent experiments (n=3). **(G)** Apoptosis detection by flow cytometry with dual annexin V-PI cell labeling. PBMCs from control (n=7) and patient (n=3) were collected and apoptosis was induced and uninduced by staurosporine. Data is represented in a scatter plot (+ median). Statistical analysis was performed using Student’s t-test for PI^-^ control PBMCs vs PI^-^ patient PBMCs in media, and PI^+^ control PBMCs vs PI^+^ patient PBMCs in staurosporine. n=7 for control and n=3 for patient. * p ≤ 0.05. Upper left quadrant (Q1), necrotic cells; upper right quadrant (Q2), late apoptotic or necrotic cells; lower left quadrant (Q3), intact cells; lower right quadrant (Q4) early apoptotic cells. The number in each quadrant refers to the percentage of cells in each subpopulation. The figure is representative of 10,000 cells analyzed independently in seven volunteers and a triplicate of patient cells. RPMI, Roswell Park Memorial Institute Medium; SDS, Sodium dodecyl Sulfate; LPS, lipopolysaccharide; Pam3c, Pam3CysSerLys4; PVDF, polyvinylidene fluoride.

Given that the atypicality of the clinical course was suggestive of underlying immunodeficiency, whole exome sequencing was performed. The sequencing revealed two heterozygous rare variants of TRAP1 on chromosome 16p13 (GRCh37): one at g.3739109 C>G (exon 3) resulting in amino acid switch from Glutamate-to-Glutamine at protein position 93 (E93Q) (**Figure 1A**) and another at g.3740885 C>T (exon 2) resulting in alanine-to-threonine switch at position 64 (A64T) (**Figure 1A**). Both E93Q and A64T SNPs lie in the N-terminal ATP-binding domain (NTD) of TRAP1 (4) and were predicted to be deleterious *in-silico* (**Figure 1B**; **Supplementary Table S1**).

### Mutant TRAP1 increased apoptosis through caspase activation

Western blot and qRT-PCR analysis of the patient’s peripheral blood mononuclear cells (PBMC) revealed diminished TRAP1 expression and transcript (**Figure 1C**, **Supplementary Figure S1B**). TRAP1 had previously been shown to protect against apoptosis (5, 6). In line with those data, we observed increased cleaved, active caspase-3 and caspase-7 in the patient’s cells, which may be associated with an overall decline in TRAP1 expression (**Figure 1C**). Caspase-3 mediates apoptosis by activating Cyclophilin D (CypD), an isomerase that regulates the mitochondrial permeability transition pore. This pore allows the release of pro-apoptotic factors from the mitochondria into the cytoplasm, initiating mitochondria-mediated apoptosis (7). Indeed, CypD was accentuated (**Figure 1C**), in line with caspase-3 augmentation in the patient’s cells.

As caspase 3 can co-activate caspase-1, the downstream mediator of inflammasome activation that leads to interleukin-1 beta (IL-1β) secretion, we stained for cleaved caspase 1 and IL-1β (8). We observed increased caspase-1 cleavage and mature IL-1β in the patient’s cells treated with TLR1/2 or TLR4 ligands, suggesting that the mutant TRAP1s exacerbate inflammasome activation (**Figures 1C, E**, **Supplementary Figure S1C**). However, the aggravated apoptosis exerted a limited inhibitory effect on the NFκB complex (9) through NFκB2, NFκB1, and RelB (**Figure 1D**).

When plasmids containing WT or mutant TRAP1 (E93Q, A64T, or the double E93Q/A64T mutant) were transfected into A549 cells, any mutant, whether single or double, was expressed lower compared to the WT protein itself. The mutants re-capitulated the immunotypic phenotype as was observed in the patient: enhanced cleaved caspase-3, caspase-7, and CypD. Of note, enhanced caspase activity was more evident in E93Q over A64T in line with in-silico prediction, and an additive effect was observed with co-expressed E93Q-A64T double mutant (**Figure 1E**). As was observed in patient cells, mutant TRAP1 reduced NFκB signaling, with the most prominent inhibitory effect on NFκB1, RelB and p65 activity observed in double E93Q/A64T mutant.

### E93Q/A64T cells exhibited reduced viability to *P. jiroveci* infection

As the presentation with PjP suggested that the effects of mutant TRAP1 was exacerbated during PjP infection, we infected TRAP1-transfected A549 human lung cells with *P. jiroveci*. Compared against WT-TRAP1, cells expressing the A64T or E93Q variant displayed reduced viability to *P. jiroveci* infection. In particular, dual E93Q/A64T-transfected cells exhibited highest cell loss (>80%) (**Figure 1F**).

### Patient’s CD4^+^ lymphocytopenia could be accounted by increased apoptosis

The increased apoptotic markers found in patient PBMCs in the presence of idiopathic CD4^+^ lymphocytopenia suggest that CD4^+^ T cells are especially sensitive to TRAP1 dysfunction. To confirm this hypothesis, patient cells were treated with staurosporine to induce apoptosis. At rest and 6 hours after staurosporine treatment, the patient’s PBMC gated on CD4^+^ cells showed elevated annexin-V-positive, propodium iodide(PI)-negative cells in the apoptotic stage compared to healthy controls. Following staurosporine, the patient’s annexin-V-positive, PI-positive cells marking cell death significantly doubled (**Figure 1G**). Collectively, our results suggest that the patient’s CD4^+^ lymphopenia was a result of increased apoptosis.

### TRAP1 E93Q/A64T-overexpressing cells and patient’s cells exhibited increased ROS

**Figure 2:**
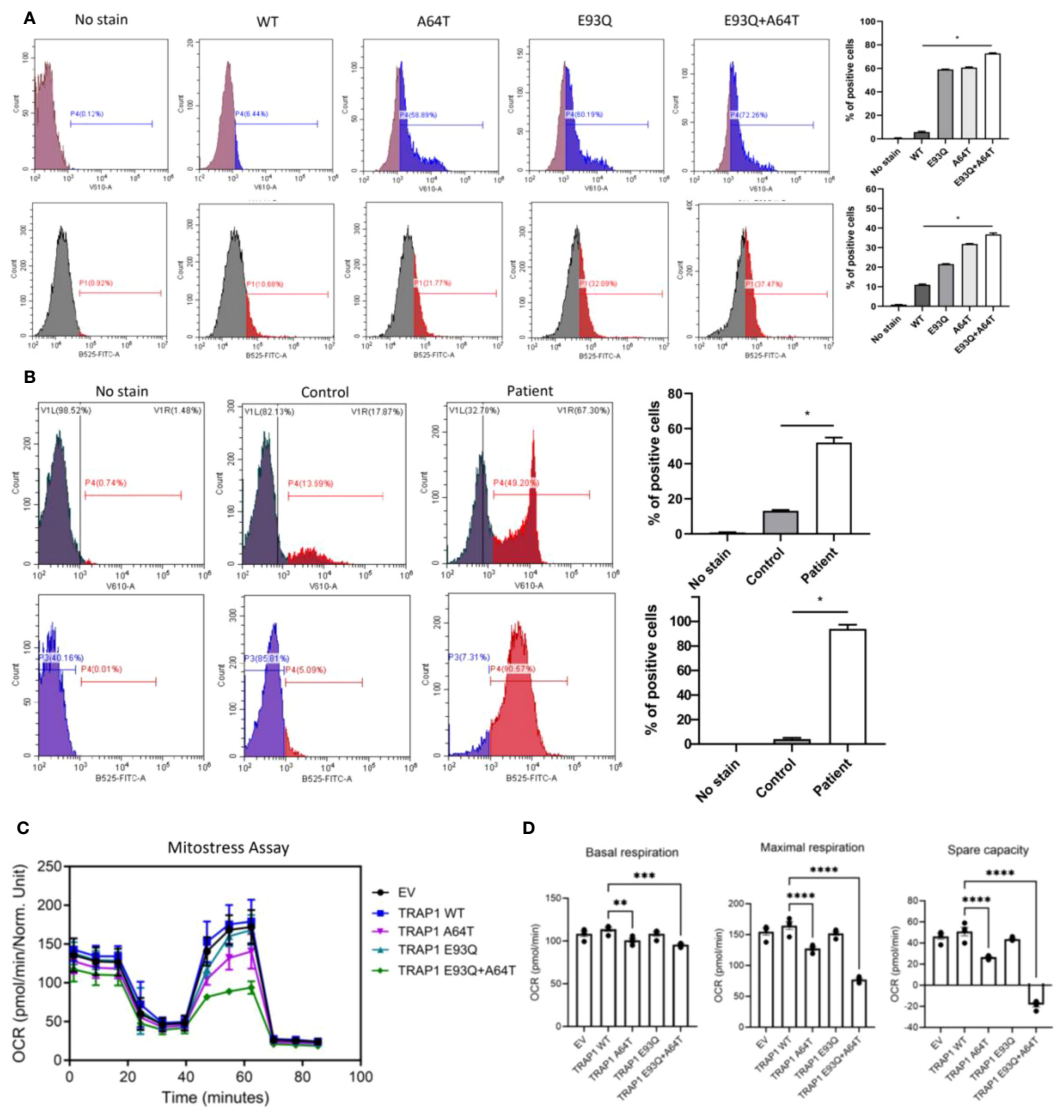
The dysregulation of TRAP1 resulted in a defective ETC, resulting in electron leakage and ROS production. **(A)** The mitochondrial and total ROS were assessed by flow cytometry in A549 cells transfected with WT and variants (A64T, E93Q, and A64T+E93Q). Representative histograms of flow cytometric analysis of MitoSOX and H2DCFDA fluorescence in different experimental groups (n=3). The bar graphs show the percentage of cells positive for mitochondrial ROS and total ROS production. The significance of the results was determined by ANOVA. * p ≤ 0.05. **(B)** The level of mitochondrial and total ROS in PBMCs from controls and the patient was measured by flow cytometry. Results are expressed as means ± standard error of the mean (SEM). n=3 per group. * p ≤ 0.05 **(C)** Oxygen consumption rate (OCR) trace of the Seahorse Mito Stress assay on A549 transfected with different Trap1 variants. (n = 3 repeats, 5 technical replicates each repeat). **(D)** Maximal glycolytic rate of A549 transfected with different Trap1 variants. (n = 2 repeats, 5 technical replicates each repeat). Results are expressed as means ± standard error of the mean (SEM). The significance of the results was determined by ANOVA (**p ≤ 0.01, *** p≤ 0.001, **** p ≤ 0.0001). ETC, Electron transport chain; ROS, Reactive Oxygen Species.

As TRAP1 regulates mitochondrial oxidative phosphorylation (OXPHOS) and suppresses ROS production, we next evaluated the effect of TRAP1 mutation on ROS production (10). Total ROS production, as measured by 2′,7′-dichlorofluorescein, was incrementally elevated in mutant-TRAP1 cells relative to WT (**Figure 2A**; top panel). Specifically, when stained with mito-SOX which probed specifically for mito-ROS, the frequency and median fluorescence intensity of E93Q- and A64T-transfected cells increased relative to WT (**Figure 2A**; bottom panel). Notably, dual-E93Q/A64T cells had 6-fold increased mito-ROS (**Figure 2A**; bottom panel). Similarly, the patient’s PBMC demonstrated markedly increased total and mito-ROS (**Figure 2B**).

### TRAP1 E93Q/A64T impaired mitochondrial respiration and glycolysis

As previous studies have shown the involvement of TRAP1 in regulating OXPHOS and glycolysis, Seahorse Mito Stress and glycolytic rate assays were performed on A549 cells transfected with the respective TRAP1 variants (10). Compared to cells transfected with WT-TRAP1 or empty vector, the double E93Q/A64T mutant expressing cells had the most markedly reduced mitochondrial respiration over the single mutants, as measured by the Mito-Stress assay (**Figure 2C**). To determine if the OXPHOS defects were due to electron transport chain (ETC) defects, we ran a BN-PAGE to look at the ETC complexes. There were no differences, suggesting that TRAP1 altered the efficiencies of ETC complexes rather than their assembly (**Supplementary Figure S2**). Similar to the observed decrease in mitochondrial respiration, the double mutant displayed a guarded reduction in the ability to increase glycolysis when the electron transport chain was blocked (**Figure 2D**).

## Discussion

Our study identifies a novel pathway involving the mitochondrial chaperon TRAP1 that confers susceptibility to opportunistic PjP infection. The TRAP1 E93Q/A64T mutation observed in our patient displayed an immunophenotype characterized by increased caspase activity alongside CypD expression and inflammasome activation, leading to enhanced apoptosis. Studies in cancer have shown that TRAP1 is a heat shock protein (HSP) that regulates the metabolic shift between glycolysis and oxidative phosphorylation (OXPHOS). Dysregulated TRAP1 results in ROS production (11). Notably, *E93Q/A64T* mutant TRAP1 led to higher ROS production in the patient’s cells with lower OXPHOS. The increased ROS induced apoptosis, including CD4 lymphocytopenia (12). The immunosuppression was compounded by elevated caspase-3 inhibition of NFkB and facilitated opportunistic PjP infection (13), which by itself has been observed to induce apoptosis in the setting of a *Pneumocystis* animal infection model (14).

There are two possible explanations for the increase in mito-ROS in cells with mutant TRAP1. As mito-ROS is generated from inefficient electron flux through the ETC, mutant TRAP1 resulted in defective ETC, leading to electron leak and ROS production. The other possibility is a reduction in GSH (reduced glutathione) production. TRAP1 over-expression yielded higher levels of GSH which quenched ROS and induced resistance to apoptosis (6). It is hypothesized that cells with attenuated TRAP1 do not produce as much GSH and therefore have lower ROS buffering capacity, as seen here in TRAP1 E93Q/A64T cells and the patient.

A perceivable limitation of the study lies in the strategy of using a clinical case with extreme presentation to elucidate a novel disease mechanism and, in turn, the potential for generalizability of the findings of TRAP1, a member of the HSP90 family, to other patients. Our findings are well in line with current knowledge that HSPs are molecular chaperons with a protective role in cell survival especially during stress, which have been previously described to interfere with programmed cell death machinery and apoptosis *in-vitro* (15, 16). Extending from TRAP1, mutations in other HSPs may potentially lead to immunodeficiency. Mutations in mitochondrial HSP60/HSP10 complexes can lead to impaired respiration and increased ROS production, suggesting that patients with these mutations could similarly have an altered immune response (17). Given the limited access to patient clinical material, the more in-depth mechanisms had to be derived from *in vitro* assays on cell lines. Thus, as these cell lines are adapted to high nutrient and oxygen-rich environments, the mutation might manifest differently under hypoxic and necrotic circumstances such as those encountered during infection, but we were generally successful in demonstrating concurrence immunophenotypically in the patient’s cells and the cell lines.

Recently, three related subjects with homozygous mutations for MEFV p.S208C and TRAP1 p.R128H presented with familial Mediterranean fever (FMF) with no reported opportunistic infection. The elevated severity of autoinflammation compared to patients with MEFV p.S208C alone was attributed to TRAP1 mutation (18). Interestingly, similar to our patient, these subjects, and a fourth patient who bore three heterozygous TRAP1 variants (p.R316H, p.Y444N, p.R128H), displayed elevated mito-ROS with overactive inflammasome activity. This suggests that TRAP1 dysregulation leads to inflammasome activation, which would not be too surprising as disturbances to mitochondrial homeostasis leads to inflammasome activation (19). Of note, p.R128H, p.A64T, and p.E93Q all lie in the conserved NTD domain required for binding ATP for the initialization of chaperon function (20). We postulate that these mutations impair ATP binding, resulting in a dysfunctional TRAP1. Our study elucidates the role of TRAP1 through E93Q/A64T which compromises mitochondrial function, particularly during infection and stress, resulting in susceptibility to opportunistic infections. The compromised immune system, together with additional apoptotic and inflammatory stress induced by the fungi PjP, constituted the perfect storm for organ injury and respiratory failure. Further investigations may be warranted to explore the precise mechanism by which TRAP1 dysregulation caused lymphocytopenia and inflammasome activation. In conclusion, our study provides novel insights into TRAP1 mutations associated with CD4^+^ lymphocytopenia and PjP susceptibility.

## Materials and methods

### Whole exome sequencing and analysis

DNA was extracted from blood samples using established methods. The concentration and purity of the DNA were quantified and 1µg of genomic DNA was fragmented using sonication, optimized to give a distribution of 200–500 base pairs. Library preparation was done using a Kapa DNA HTP Library Preparation Kit. Hybridization of the adapter ligated DNA is performed at 47^o^C, for 64 to 72 hours, to a biotin-labelled probe included in the Nimblegen SeqCap EZ Human Exome Kit. Libraries were sequenced using the Illumina Hiseq 4000 sequencer and paired-end 151bp reads were generated for analysis. Confirmation of the identified variant of interest was performed via Sanger sequencing. For base calling, we performed alignment of the raw reads onto the GRCh37 reference genome. After the reads were mapped, we used Picard to label read groups and mark PCR duplicates followed by rearranging of Binary Alignment Map. Local realignment and recalibration were executed to calculate and adjust the quality scores to be nearer to the actual probability of mismatching the reference genome. For variant calling, the main component was HaplotypeCaller from GATK version 3. GenotypeGVCFs function to jointly aggregate multiple samples from previously single-sample haplotype calling. Sets of high-quality variants including HLA types were annotated to understand the allele frequency, type, protein consequence, domain etc. The identification of candidate genes of interest was adopted from Ambry’s clinical variant classification scheme and the American College of Medical Genetics and Genomics variant classification recommendations. The patient had no gene mutations of the IL-12/IFN-γ axis related to Mendelian susceptibility to mycobacterial disease (MSMD) or gene mutations related to the NFκB pathway.

### PBMC isolation and stimulation

Venous blood (10 ml) was collected into heparinized tubes and processed immediately. Blood was withdrawn under authorized supervision from subjects who had given their consent. PBMCs were isolated using gradient centrifugation in Ficoll-Paque solution (Ficoll-Paque PLUS, GE healthcare, UK). Isolated PBMCs were incubated in RPMI+ medium-1640 (Biowest France, modified with 10 mM pyruvate and 10 μg/ml gentamicin) with heat inactivated 10% fetal bovine serum. For the stimulation assays, the PBMCs were counted, and tested for viability using the Countess (Invitrogen, Carlsbad, CA). PBMCs seeded on round-bottom (24h) and flat-bottom (48h) 96-well plate at 5 × 10^5^ cells/well were stimulated in duplicate with *Escherichia coli* lipopolysaccharide (LPS; 10 ng/ml), Pam3Cys (10 μg/ml), heat-killed *Mycobacterium abscessus* (NTM; 1 × 10^6^ cfu/ml), heat-killed *Mycobacterium tuberculosis* (MTB; 1X10^6^/ml), and heat-inactivated *Candida albicans* (1X10^6^/ml). The supernatants were collected after centrifugation of the plate and stored at –20°C until cytokines were measured.

### ELISA

Secreted IL-6, IL-1β, and tumor necrosis factor-alpha (TNF-α) (eBioscience, San Diego, CA) were measured by a commercial ELISA kit following the instructions provided by the manufacturer.

### RNA isolation and *quantitative reverse-transcription* PCR

Total RNA was extracted from PBMCs with TRIzol reagent (Life Technologies, USA) and reverse transcribed into cDNA using an iScript™ cDNA Synthesis Kit (BIO-RAD), and the qRT-PCR was performed using GoTaq qPCR Mastermix (Promega Corporation, Madison, WI, USA) with a CFX connect (BIO-RAD) Thermocycler. The primers used to amplify transcripts of human Trap1 were 5′- AGG ACG ACT GTT CAG CAC G -3′ (forward) and 5′ -CCG GGC AAC AAT GTC CAA AAG -3′ (reverse). Beta-2-microglobulin (B2M) was used as endogenous control, for which the primers were 5′ - ATG AGT ATG CCT GCC GTG TG -3′ (forward) and 5′ -CCA AAT GCG GCA TCT TCA AAC -3′ (reverse). The relative gene expression was calculated using the 2-ΔΔCT method. Values were expressed as a ratio of fold increase to mRNA levels of the control cells.

### PjP infection and cell viability assay

*P. jiroveci* was cultured using a conventional method and infected in A549 cells in a 96-well plate and an XTT Assay for Cell Viability (Sigma) was carried out 24h and 48h after infection following the manufacturer’s protocol.

### Cloning and transfection

To generate EGFP-TRAP1 mammalian expression construct (pEGFP-TRAP1), human wild type TRAP1 cDNA was PCR amplified from a clone from the TOH7500-Human-Lentiviral-ORF-Expression-Library (TransOMIC Technologies, Inc.). EGFP-TRAP1 plasmid constructs with individual A64T (pEGFP-TRAP1-A64T), E93Q (pEGFP-TRAP1-E93Q), A64T (pEGFP-TRAP1-A64T), and E93Q (pEGFP-TRAP1-E93Q) mutations were created by overlap extension PCR protocol and assembled into XhoI – KpnI site of the pEGFP-C1 vector (Clontech) using the HiFi DNA Assembly Protocol. All clones were sequence confirmed to check whether GFP and TRAP1 were in frame and for the presence of site-specific mutation and absence of any mutations.

Transfection of empty pEGFPC1 vector (EV), pEGFP-TRAP1 (WT), pEGFP-TRAP1-A64T (A64T), pEGFP-TRAP1-E93Q (E93Q), and pEGFP-TRAP1-A64T+E93Q (A64T+E93Q) was carried out in A549 cells using LipofectamineTM 3000 Reagent (Thermo Fisher Scientific, Waltham, MA, USA) according to manufacturer’s protocol in 6-well plates. Furthermore, 24h after transfection, the cells were stimulated with LPS and Pam3C for 24h and Western blotting was performed as described.

### Western blot

PBMCs were stimulated with LPS and Pam3C for 30 min or unstimulated (RPMI). Western blotting was performed using a standard protocol. Briefly, total protein was extracted and separated by 10% SDS-PAGE, and the separated proteins were transferred onto PVDF membranes. The membranes were blocked with 5% nonfat milk at room temperature for 1h and then incubated with specific primary antibodies overnight at 4°C. The primary antibodies included antibodies against TRAP1(92345; Cell Signaling, Technology), NF-κB1 (3035; Cell Signaling, Technology), NF-κB2 (4882; Cell Signaling, Technology), RelB (10544; Cell Signaling, Technology), RelA (Santa Cruz Biotechnology), Caspase-3 (9662; Cell Signaling, Technology), Caspase-7 (9492; Cell Signaling, Technology) caspase1 (1:1000; ab286125; Abcam), IL-1β (1:500; sc7884; Santa Cruz Biotechnology), and β-actin (1:1000; sc47778; Santa Cruz Biotechnology). The membranes were incubated with appropriate secondary antibodies (anti-rabbit, and anti-mouse IgG, Cell Signalling, Technology) for 1h at room temperature, and the bands were visualized with the Clarity Western ECL blotting substrate (Bio-Rad, USA).

### Apoptosis and flow cytometry

5x10^5^ PBMCs were cultured in a 48 well plate. To induce apoptosis, cells were treated with 0.1uM of staurosporine. Unstimulated cells were incubated with 0.01% DSMO as solvent control to define the basal level of apoptotic and dead cells. Six hours later, cells were collected from the plate and stained with anti-CD3, anti-CD4, anti-CD8, and anti-CD45 antibodies (BD Bioscience). The apoptotic and dead cells were then detected using the FITC Annexin V Apoptosis Detection Kit according to the manufacturer’s instruction. The data were collected using FACScanto II and analyzed using Flowjo.

### Flow cytometry for ROS detection

Patient and control PBMCs and cells were incubated in 10 μM dichlorodihydrofluorescein diacetate (H2DCFDA), or 5 μM MitoSOX (mitochondrially targeted DHE) in phenol-free RPMI at 37°C for 30 min and flow cytometry was performed according to the manufacturer’s instructions. The data were analyzed with CytExpert software.

### Seahorse assay

A549 were transfected with the plasmids via Lipofectamine 3000. The next day, the cells were seeded at 20,000 cells/well into Seahorse XF96 Cell Culture Microplates (Agilent Technologies 101085–004). After 24h, the media were replaced with XF basal DMEM supplemented with 1 mM pyruvate (Sigma-Aldrich S8636), 2 mM glutamine (Life Technologies 25030081), and 10 mM glucose (Sigma-Aldrich G8644). The plate was incubated at 37°C with no CO2 for 45 min before it was run on the Agilent Seahorse XF Analyzer. XFe96 sensor cartridges (Agilent) were hydrated in water the night before and calibrated with Seahorse XF Calibrant (Agilent) for 2h at 37°C with no CO2. For the MitoStress assay, final concentrations of 5 μM oligomycin (Sigma-Aldrich G8644), 1 μM FCCP (Sigma-Aldrich C2920), and 1 μM Rotenone/Antimycin A (Sigma R8875; A8674) were injected sequentially. The data were collected via the Agilent Seahorse Wave software. The Seahorse assay was normalized using the citrate synthase activity in each well. After removing the media, 47.5 μl of CS buffer [200 mM Tris buffer at pH 8.0, 0.2% Triton X-100 (v/v), 10 μM DTNB (Sigma D8130), and 1 mM Acetyl-CoA (Sigma-Aldrich A2181)] were added per well. Furthermore, 2.5 μl of 10 mM oxaloacetate (Sigma O4126) was added to each well. The plate was immediately placed into the Tecan Microplate Reader M200 (Tecan). Absorbance at 412 nm at 37°C was recorded every 30s for 8 min. The relative levels of CS activity were directly proportionate to the relative rate of change in absorbance.

### Blue Native PAGE

A549 were transfected with the plasmids via Lipofectamine 3000 and harvested after 2 days. Mitochondria were isolated and ran on native gels as described (21).

### Statistical analysis

The results are presented as the mean ± standard error of the mean (SEM) for at least three independent experiments. The results of the experiments were plotted and analyzed using GraphPad Prism software. Student’s t-test was used for statistical analysis of two groups and ANOVA for three groups or more. The p values ≤ 0.05 were considered statistically significant.

## Data availability statement

The data presented in the study are deposited in the NCBI repository, with accession number PRJNA1138899.

## Ethics statement

Approval of study was granted by the National Healthcare Group Domain Specific Research Board (No.2007/00517). Written Informed consent was obtained from study subjects.

## Author contributions

AA: Methodology, Project administration, Validation, Writing – review & editing, Resources, Visualization. KP: Methodology, Project administration, Writing – review & editing. CH: Data curation, Methodology, Validation, Writing – review & editing. AJ: Data curation, Visualization, Writing – review & editing. JH: Methodology, Project administration, Visualization, Writing – review & editing. SR: Visualization, Writing – review & editing. QS: Methodology, Project administration, Writing – review & editing. RT: Methodology, Writing – review & editing. ZT: Data curation, Writing – review & editing. DK: Validation, Visualization, Writing – review & editing. LN: Data curation, Writing – review & editing. SP: Visualization, Writing – review & editing. RF: Methodology, Visualization, Writing – review & editing. CC: Visualization, Writing – review & editing. JZ: Methodology, Validation, Visualization, Writing – review & editing. CL: Methodology, Supervision, Visualization, Writing – review & editing. LC: Funding acquisition, Investigation, Methodology, Project administration, Supervision, Validation, Visualization, Writing – original draft, Writing – review & editing, Resources.
