# Supplementary material for: Mitochondrial chaperon TNF-receptor- associated protein 1 as a novel apoptotic regulator conferring susceptibility to Pneumocystis jirovecii pneumonia
Source: Front Immunol. 2024 Aug 19;15:1423086. doi: 10.3389/fimmu.2024.1423086 (PMC11368041; doi:10.3389/fimmu.2024.1423086)

Supplementary Material

***Mitochondrial Chaperon TNF-Receptor-Associated Protein 1 as a Novel Apoptotic Regulator Conferring Susceptibility to Pneumocystis jirovecii Pneumonia***

Aseervatham Anusha Amali^1^†, Kathirvel Paramasivam^2^†, Chiung Hui Huang^3^, Abhinav Joshi^4^, Jayshree L. Hirpara^11^, Sharada Ravikumar^1^, Qi Hui Sam^1,6^, Rachel Ying Min Tan^1^, Zhaohong Tan^1^, Dilip Kumar^12^, Leonard M. Neckers^4^, Shazib Pervaiz^5^, Roger Foo^7,8^, Candice Chan^9^, Jing Zhu^2^, Cheryl Lee^10‡^, Louis Yi Ann Chai^1,6,8‡^*

*** Correspondence:** Corresponding Author: [chailouis@hotmail.com](mailto:chailouis@hotmail.com)

# Supplementary Table 1. Characteristics of Identified Putative Complex Heterozygote TRAP1 Rare Variants E93Q and A64T on chromosome 16p13 (GRCh37)

| Variant | **p.E93Q** | **p.A64T** |
| --- | --- | --- |
| gDNA position | g.3739109 | g.3740885 |
| Nucleotide change | cytosine (C) to guanine (G) | cytosine (C) to thymine (T) |
| Amino acid change | glutamic acid (E) to glutamine (Q) | alanine (A) to threonine (T) |
| dbSNP | rs558631874 | rs368129763 |
| Alternate allele frequency [gnomAD] | 0.00039786% | 0.0039094% |
| Conservation | moderately conserved nucleotide (phyloP: 7.20) | weakly conserved nucleotide (phyloP: 3.49) |
| Coding effect | missense | missense |
| SIFT (v.6.2.0) | deleterious (score: 0.00) | TOLERATED (score: 0.11) |
| Polyphene2 | probably damaging (score: 1) | benign (score: 0.222) |
| CADD (v1.6) | Phred: 26.5 | Phred: 16.25 |

dbSNP - Single Nucleotide Polymorphism Database, gnomAD - Genome Aggregation Database, SIFT - Sorting Intolerant From Tolerant, PolyPhen-2 - Polymorphism Phenotyping v2, CADD - Combined Annotation Dependent Depletion

## Supplementary Figures

Supplementary Figure 1. **Supplementary Figure 1.** (**A**) Clinical course and investigation of the patient. (**B**) qRT-PCR analysis showing reduction of Trap1 expression in patient (n=3). (**C**) PBMC from patient and controls were stimulated with lipopolysaccharide (10 µg/mL), Pam3Cys (10 µg/mL) in RPMI+ medium. The supernatants were collected and the secretion of TNF-α, IL-1β, and IL-6 were measured by ELISA. The results were pooled from up to 3 experiments. * p ≤ 0.05. qRT-PCR, Quantitative reverse transcription polymerase chain reaction.

##
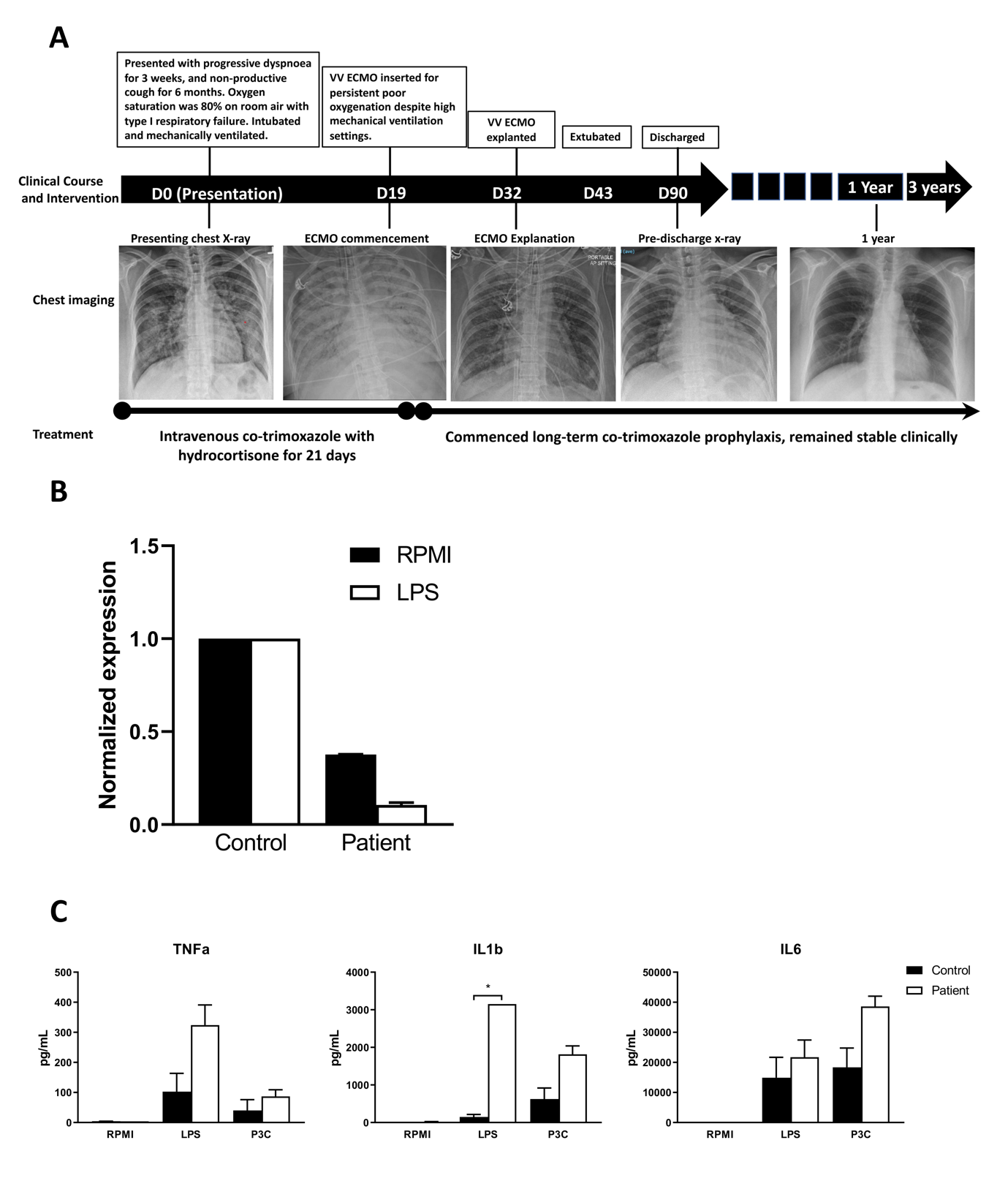


**Supplementary Figure 2. BN-PAGE showing mitochondrial complexes and super complexes.**


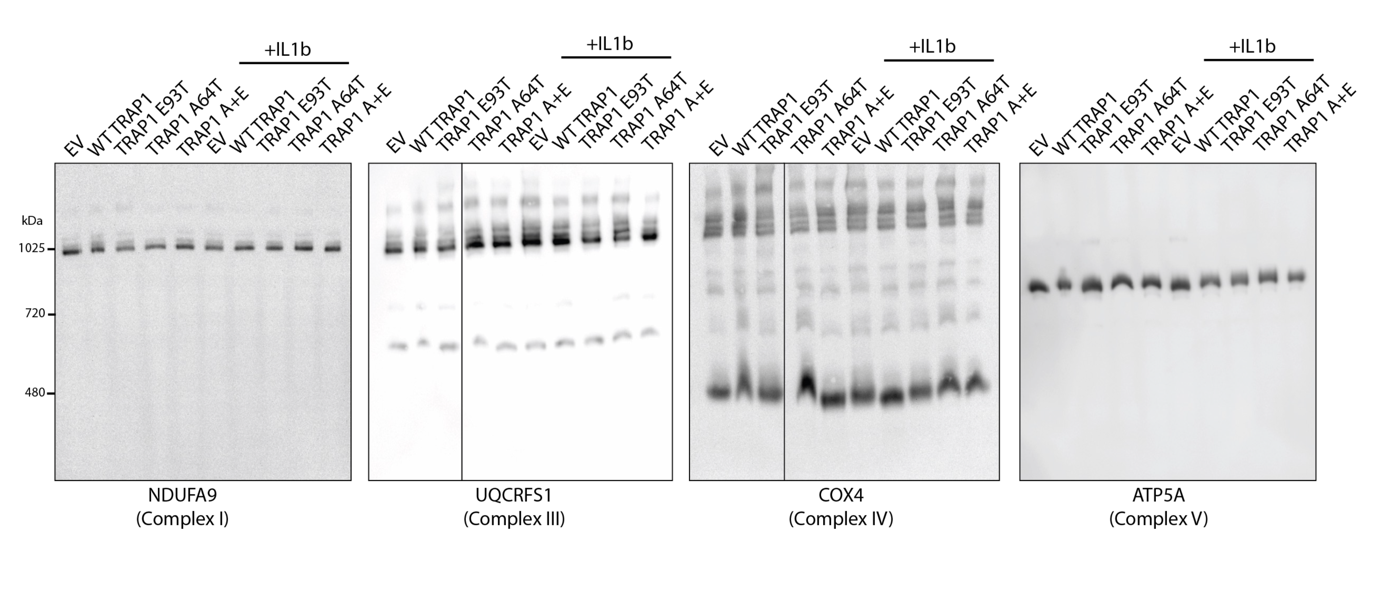

Supplement: Supplementary file 1 [file DataSheet_1.docx]
